# IKK inhibition by BMS-345541 suppresses breast tumorigenesis and metastases by targeting GD2^+^ cancer stem cells

**DOI:** 10.18632/oncotarget.16294

**Published:** 2017-03-16

**Authors:** Venkata Lokesh Battula, Khoa Nguyen, Jeff Sun, Mary Kathryn Pitner, Bin Yuan, Chandra Bartholomeusz, Numsen Hail, Michael Andreeff

**Affiliations:** ^1^ Department of Leukemia, Section of Molecular Hematology and Therapy, University of Texas MD Anderson Cancer Center, Houston, TX, USA; ^2^ Department of Breast Medical Oncology, University of Texas MD Anderson Cancer Center, Houston, TX, USA

**Keywords:** breast cancer, cancer stem cells, GD2, GD3 synthase, NFκB

## Abstract

We have identified that the ganglioside GD2 is a marker for breast cancer stem cells (BCSCs), and that targeting the enzyme GD3 synthase (GD3S, which regulates GD2 biosynthesis) reduces breast tumorigenesis. The pathways regulating GD2 expression, and their anomalous functions in BCSC, are unclear. Proteomic analysis of GD2^+^ and GD2^-^ cells from breast cancer cell lines revealed the activation of NFκB signaling in GD2^+^ cells. Dose- and time-dependent suppression of NFκB signaling by the small molecule inhibitor BMS-345541 reduced GD2^+^ cells by > 90%. Likewise, BMS-345541 inhibited BCSC GD3S expression, mammosphere formation, and cell migration/invasion *in vitro*. Breast tumor-bearing mice treated with BMS-345541 showed a statistically significant decrease in tumor volume and exhibited prolonged survival compared to control mice, with a median survival of 78 d for the BMS-345541-treated group vs. 58 d for the controls. Moreover, in an experimental metastases model, treatment with BMS-345541 reduced the lung metastases by > 5-fold. These data suggest that GD2 expression and function, and NFκB signaling, are related, and they control BCSCs tumorigenic characteristics. Thus, the suppression of NFκB signaling by BMS-345541 is a potentially important advance in controlling breast cancer growth and metastases.

## INTRODUCTION

Breast cancer stem cells (BCSCs) are a subpopulation of primary breast tumor cells that are resistant to most conventional chemotherapeutic agents, and hence they can cause secondary primary tumors if they are not eradicated from the body via surgery or radiotherapy [1, 2]. These cells can also cause tumor metastasis through their ability to dissociate from the primary tumor and invade vicinal tissues and vasculature [3]. Because of their quiescent nature, BCSCs are difficult to target in general [4], and molecular characteristics that are specific for these cells are in desperate need of identification.

Recently, our group identified the ganglioside GD2 as a marker for BCSCs [5]. GD2 is co-expressed on well-established CD44^high^CD24^low^ cells, and GD2^+^ cells can form mammospheres in low-adherent conditions, migrate, and invade *in vitro*. They can also initiate tumors from as few as 10 cells *in vivo*. We also found that GD3 synthase (GD3S), an enzyme that synthesizes GD3 that is a precursor molecule for GD2, was highly expressed on GD2^+^ breast cancer cells [5]. Genetic and pharmacologic inhibition of GD3S reduced GD2 expression and inhibited tumor growth *in vivo*. Inhibition of GD3S expression also reduced lung metastases of breast cancer cells in immunodeficient mice [6].

We also recently reported that the overexpression of the NFκB (p65) super-repressor inhibited GD3S expression in breast cancer cells [6]. NFκB is activated via “canonical” and “non-canonical” pathways. The canonical pathway is triggered by pro-inflammatory cytokines such as TNFα and IL-1. The non-canonical NFκB pathway is activated by TNF family cytokines [7, 8], CD40 ligand [7], B-cell activating factor (BAFF/TNFSF13B) [9], and receptor activator of NFκB ligand (RANKL/TNFSF11) [10] resulting in the activation of RelB/p52 complexes [9]. NFκB is a known regulator of many aberrant cellular functions in cancer cells, including cell migration, chemo-resistance, and tumor engraftment [11–13] Yamamoto *et al*., reported that NFκB non-cell autonomously regulated cancer stem cells in basal-like breast cancer, suggesting a major role of this signaling pathway in BCSC function [14].

To date, the signaling pathways regulating GD2 expression and their anomalous functions in BCSC are unclear. In this manuscript, we show that NFκB signaling is activated in GD2^+^ BCSCs and that the inhibition of this signaling by the IKKα/β inhibitor BMS-345541 suppresses BCSC tumorigenic functions, including metastasis. In addition, we identify BMS-345541 as a potential therapeutic agent to inhibit the spontaneous generation of GD2^+^ BCSCs, which could conceivably inhibit breast cancer growth and metastases.

## RESULTS

### Proteomic profiling revealed activation of NFκB signaling in GD2^+^ compared to GD2^-^ cells

The signaling pathways regulating GD3S and GD2 expression are unclear. To identify potential components of these pathways, GD2^+^ and GD2^-^ cells from MDA-MB-231 and SUM159 breast cancer cell lines were sorted using FACS, and the cell lysates were assayed on a Kinexus antibody microarray. A total of 850 different validated proteins, belonging to various signaling pathways, were assayed in each sample. The proteins that were differentially expressed or phosphorylated in the GD2^+^ and the GD2^-^ SUM159 (Table 1) and MDA-MB-231 cells (supplementary Table 1) were organized based on their z-scores [15]. Pathway analysis of the differentially expressed and phosphorylated proteins using Ingenuity pathway analysis tool revealed the activation of NFκB, STAT3, focal adhesion kinase (FAK), p53, platelet-derived growth factor β (PDGFβ), and p38 MAPK signaling pathways, among others, in the GD2^+^ cells (Supplementary Figure 1).

**Table 1:** Proteins up- or down-regulated in GD2+ compared with GD2- cells

Target Protein Name	Phospho Site (Human)	Full Target Protein Name	Z-ratio (SUM159-GD2+, SUM159-GD2-)
Caveolin 2	Pan-specific	Caveolin 2	3.12
Cyclin E	T395	Cyclin E1	1.89
Csk	Pan-specific	C-terminus of Src tyrosine kinase	1.89
p38a MAPK	Pan-specific	Mitogen-activated protein-serine kinase p38 alpha	1.82
STAT2	Pan-specific	Signal transducer and activator of transcription 2	1.80
S6K	S411	p70 ribosomal protein-serine S6 kinase	1.78
CDK1 (CDC2)	Pan-specific	Cyclin-dependent protein-serine kinase 1	1.76
PKR1	T446	Double-stranded RNA-dependent protein-serine kinase	1.73
RelB	S573	Transcription factor RelB	1.67
PKG1	Pan-specific	Protein-serine kinase G1 (cGMP-dependent protein kinase)	1.67
p25	Pan-specific	CDK5 regulatory subunit p25	1.51
Pyk2	Pan-specific	Protein-tyrosine kinase 2	1.40
IKKa	T23	Inhibitor of NF-kappa-B protein-serine kinase alpha (CHUK)	1.31
Cofilin 1	Pan-specific	Cofilin 1	1.26
YSK1	Pan-specific	Serine/threonine-protein kinase 25	1.25
PKCh	T655	Protein-serine kinase C eta	1.24
Cbl	Y700	Signal transduction protein CBL	1.23
MDM2	S166	double minute 2	1.21
NFKB p65	S536	NF-kappa-B p65 nuclear transcription factor	1.18
hHR23B	Pan-specific	UV excision repair protein RAD23 homolog B	1.17
anti-actin	Pan-specific	Actin	1.15
RSK1	Pan-specific	Ribosomal S6 protein-serine kinase 1	1.12
Smad2	T200	Mothers against decapentaplegic homologs 2	1.05
Cdc34	Pan-specific	Cell division cycle 34 (ubiquitin-conjugating ligase)	1.05
PKCe	Pan-specific	Protein-serine kinase C epsilon	1.02
Hsp90a/b	Pan-specific	Heat shock 90 kDa protein alpha/beta	1.01
Hsp60	Pan-specific	Heat shock 60 kDa protein 1 (chaperonin, CPN60)	-1.02
mMOB1	Pan-specific	Preimplantation protein 3	-1.03
Histone H2B	S15	Histone H2B	-1.03
JAK2	Y1007+Y1008	Janus protein-tyrosine kinase 2	-1.06
Histone H3	S29	Histone H3.3	-1.08
JAK3	Pan-specific	Janus protein-tyrosine kinase 3	-1.09
c-IAP1	Pan-specific	Cellular inhibitor of apoptosis protein 1 (baculoviral IAP repeat-containing protein 3, apoptosis inhibitor 2 (API2))	-1.10
Bcl-xS/L	Pan-specific	Bcl2-like protein 1	-1.12
Bcl-xL	Pan-specific	Bcl2-like protein 1	-1.13
KAP	Pan-specific	Cyclin-dependent kinase associated phosphatase (CDK inhibitor 3, CIP2)	-1.15
p107	Pan-specific	Retinoblastoma (Rb) protein-related p107 (PRB1)	-1.16
Catenin b1	Pan-specific	Catenin (cadherin-associated protein) beta 1	-1.18
EGFR	Y1172	Epidermal growth factor receptor-tyrosine kinase	-1.19
Hsp90a/b	Pan-specific	Heat shock 90 kDa protein alpha/beta	-1.21
GFAP	S8	Glial fibrillary acidic protein	-1.22
GRK2 (BARK1)	Pan-specific	G protein-coupled receptor-serine kinase 2	-1.25
Hsp27	S15	Heat shock 27 kDa protein beta 1 (HspB1)	-1.27
Cyclin A	Pan-specific	Cyclin A1	-1.29
Erk1 + Erk2	T202	Extracellular regulated protein-serine kinase 1 (p44 MAP kinase)+Extracellular regulated protein-serine kinase 2 (p42 MAP kinase)	-1.30
EGFR	Pan-specific	Epidermal growth factor receptor-tyrosine kinase	-1.31
IkBb	Pan-specific	Inhibitor of NF-kappa-B beta (thyroid receptor interacting protein 9)	-1.47
CDK9	Pan-specific	Cyclin-dependent protein-serine kinase 9	-1.49
Fos	Pan-specific	Fos-c FBJ murine osteosarcoma oncoprotein-related transcription factor	-1.81
PP2B/Aa	Pan-specific	Protein-serine phosphatase 2B - catalytic subunit - alpha isoform	-1.83
IkBa	Pan-specific	Inhibitor of NF-kappa-B alpha (MAD3)	-2.27

Protein lysates from SUM159 cells were analyzed on antibody microarrays (Kinexus). Differentially expressed or activated proteins are listed. Z score transformation provides a way of standardizing data across a wide range of experiments and allows the comparison of independent datasets. Data normalized by Z score transformation can be used directly in the calculation of statistically significant changes in gene expression between different samples. Z-ratio given below are obtained using methods described before [15].

**Figure 1:**
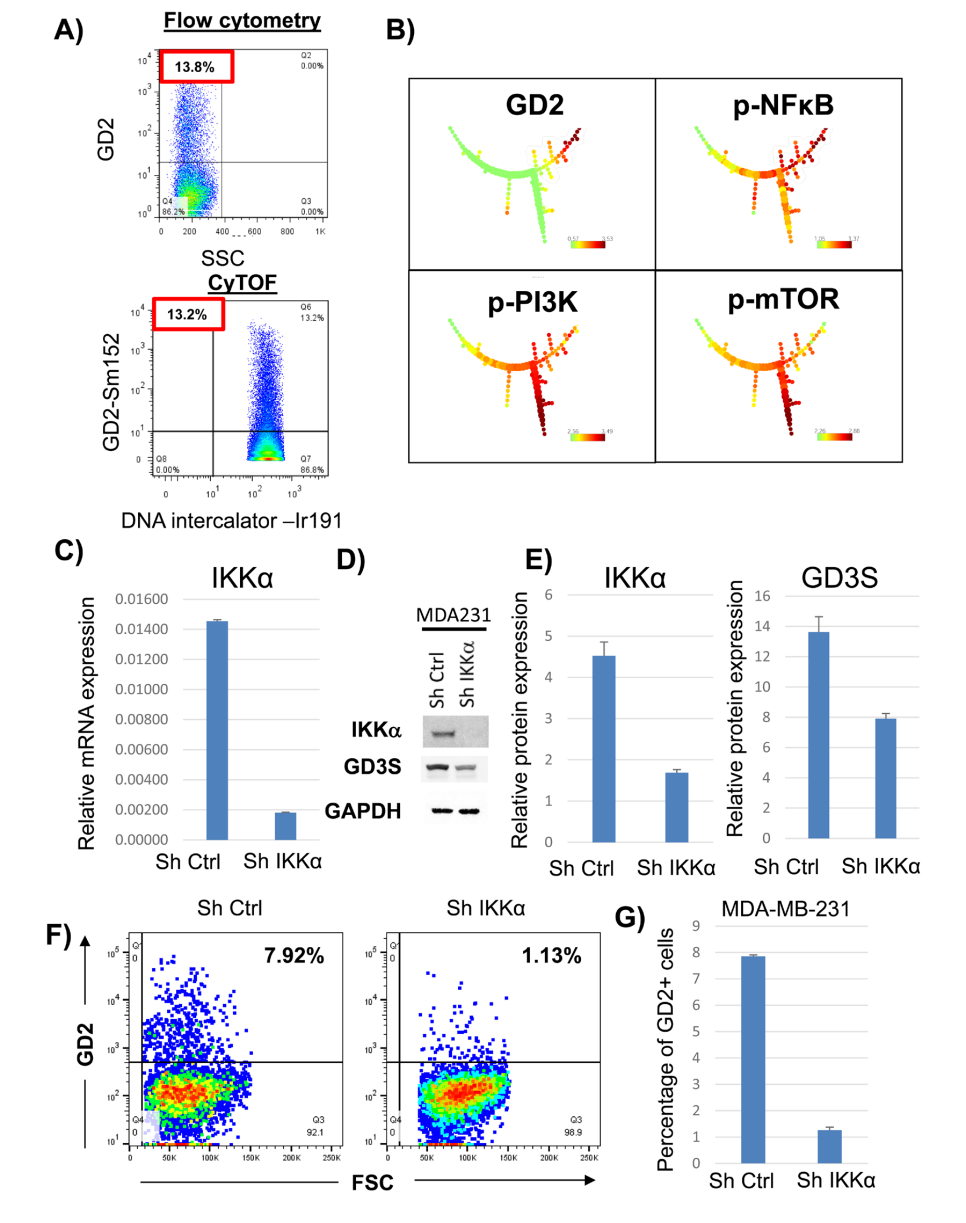
Activation of NFκB signaling in GD2^+^ cells **A**. MDA-MB-231 cells were stained with anti-GD2 conjugated with phycoerythrin (fluorochrome) or Sm152 (metal) and analyzed on an LSR II flow cytometer or by CyTOF, respectively. The data were analyzed on FlowJo software. **B**. MDA-MB-231 cells were incubated with anti-GD2 antibody conjugated with Sm152, anti-pNFκB(p65) conjugated with Sm149, anti-pPI3K(Y607) antibody conjugated with Gd158, and anti-pmTOR(S248) antibody conjugated with Dy164. All the samples were co-stained with and iridium (DNA-intercalator) conjugated with Rh103 for gating. The cells were analyzed on by CyTOF. The data were analyzed using the SPADE complex data analysis tool. A gradient from green (low) to red (high) indicates expression of each marker in different populations in the SPADE tree structure.. **C**. qRT-PCR analysis of IKKα in control and IKKα knockdown MDA-MB-231 cells. **D**. Western blot analysis to determine IKKα and GD3S protein expression in control and IKKα knockdown MDA-MB-231 cells. **E**. Bar graph represents quantification of IKKα and GD3S protein expression in control and IKKα knockdown MDA-MB-231 cells. GAPDH served as loading control **F**. Flow cytometry analysis was performed to measure GD2 expression in control and IKKα knockdown MDA-MB-231 cells. **G**. Bar graph represents the percentage of GD2^+^ cells in control and IKKα knockdown MDA-MB-231 cells.

**Figure 2:**
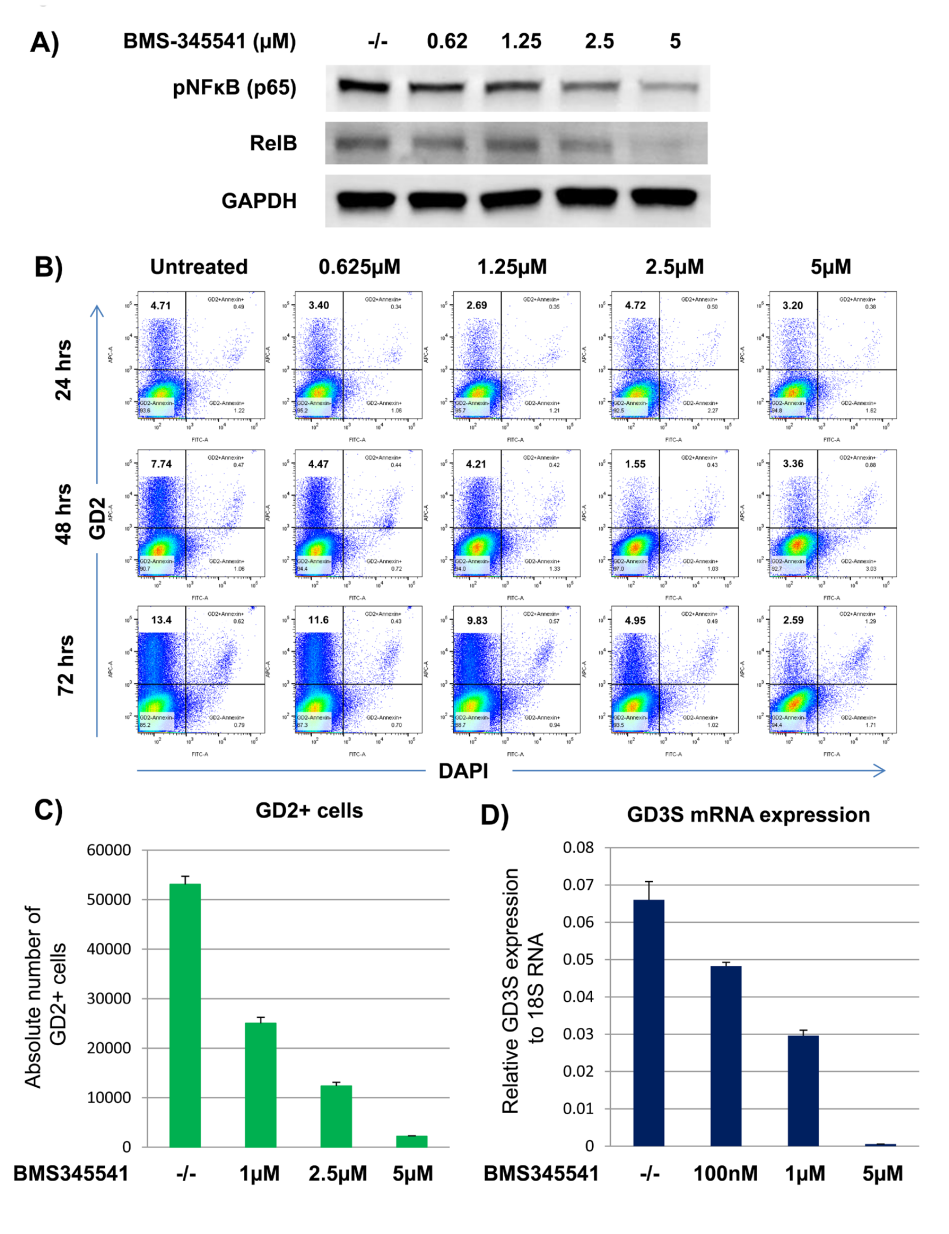
BMS-345541 inhibits GD2 and GD3S expression by inhibiting NFκB signaling in breast cancer cells A) MDA-MB-231 cells were treated with or without varying concentrations of BMS-345541 for 24 h. For the western blot, the cell lysates were run on a gel, and the transfer membranes were incubated with anti-pNFκB or anti-RelB antibodies. GAPDH served as a loading control. **B**. MDA-MB-231 cells were treated with BMS-345541 at concentrations of 1, 2.5, and 5 μM for 72 h. The cells were then stained with anti-GD2 antibody conjugated with allophycocyanin and analyzed on an LSR II flow cytometer. The cells were also stained with DAPI to exclude dead cells. **C**. The bar graph represents the absolute number of GD2^+^ cells from the experiment in Figure 2A. The assay was performed using TruCount absolute counting beads. **D**. GD3S mRNA expression analysis by quantitative RT-PCR. MDA-MB-231 cells were treated with BMS-345541 at concentrations of 0.1, 1, or 5 µM for 24 h. Relative GD3S mRNA expression levels were analyzed by TaqMan quantitative RT-PCR using the 7900HT fast real-time polymerase chain reaction system. 18S RNA served as the equal loading control.

To validate the antibody array data, we performed CyTOF. MDA-MB-231 cells were stained with antibodies to identify both extra-cellular and intra-cellular targets. CyTOF identified a fraction (i.e., 13% ± 2%) of GD2^+^ cells equivalent to that observed (i.e., 14 ± 2.5%) using conventional flow cytometry (Figure 1). The multi-parameter CyTOF data generated from MDA-MB-231 cells were analyzed by SPADE software [16]. Figure 1B shows the tree structures generated by SPADE analysis, revealing that pPI3K and pmTOR were down-regulated in the GD2^+^ cells. Interestingly, pNFκB was up-regulated in GD2^+^ cells compared with their GD2^-^ counterparts. Next, to investigate if GD2 and GD3S expression was dependent on NFκB signaling, we knocked down IKKα (a crucial factor in mediating canonical and non-canonical NFκB signaling), in MDA-MB-231 using shRNA (Figure 2C). mRNA and protein expression analysis validated inhibition of IKKα expression by 70 to 90% in IKKα knock-down, compared to control, cells (Figure 2C through 2E). Interestingly, GD3S expression was downregulated ~ 40% in the IKKα knockdown compared to controls (Figure 2D and 2E). More importantly, flow cytometry analysis revealed > 80% decrease in the percentage of GD2^+^ cells in IKKα-KD cells compared to the control cells (Figure 2F and 2G). These data suggest that NFκB signaling is activated in GD2^+^ cells and inhibition of this pathway by IKKα knockdown inhibits GD2 and GD3S expression in breast cancer cells.

### NFκB inhibition by BMS-345541 suppresses tumorigenic and metastatic characteristics in BCSCs

We recently reported that the overexpression of the NFκB (p65) super-repressor inhibited GD3S expression in breast cancer cells [6], suggesting that NFκB signaling could be an important regulator in BCSC tumorigenic functions. To examine this further, we tested several small-molecule NFκB signaling inhibitors, including JSH-23, wedelolactone, and BMS-345541 to determine their effect on BCSC functions. We found that BMS-345541 was very effective. Treatment of MDA-MB-231 cells with this agent inhibited canonical pathway-associated proteins, including pNFκB (p65), as well as non-canonical pathway-associated proteins, including RelB, in a dose-dependent manner (Figure 2A).

To test whether BMS-345541 could also inhibit GD2^+^ cell growth, MDA-MB-231 and SUM159 cells were treated with BMS-345541 at various concentrations (i.e., 0.625, 1.25, 2.5, and 5 µM) or an equal volume of the vehicle PBS (control) for 24, 48, or 72 h. Treatment with BMS-345541 decreased the percentage of GD2^+^ MDA-MB-231 cells from 12.5% ± 3% to 1.5% ± 1%, and it inhibited the absolute number of GD2^+^ cell numbers by > 90% in a dose- and a time-dependent manner (Figure 2B and 2C). We have reported that GD2 expression is tightly regulated by GD3S in BCSCs [5]. Therefore, we next tested whether BMS-345541 could inhibit GD3S expression at the transcription level. In MDA-MB-231 cells treated with BMS-345541, GD3S mRNA expression was dramatically inhibited, by > 98% (Figure 2D), indicating that GD3S expression is highly dependent on active NFκB signaling.

**Figure 3:**
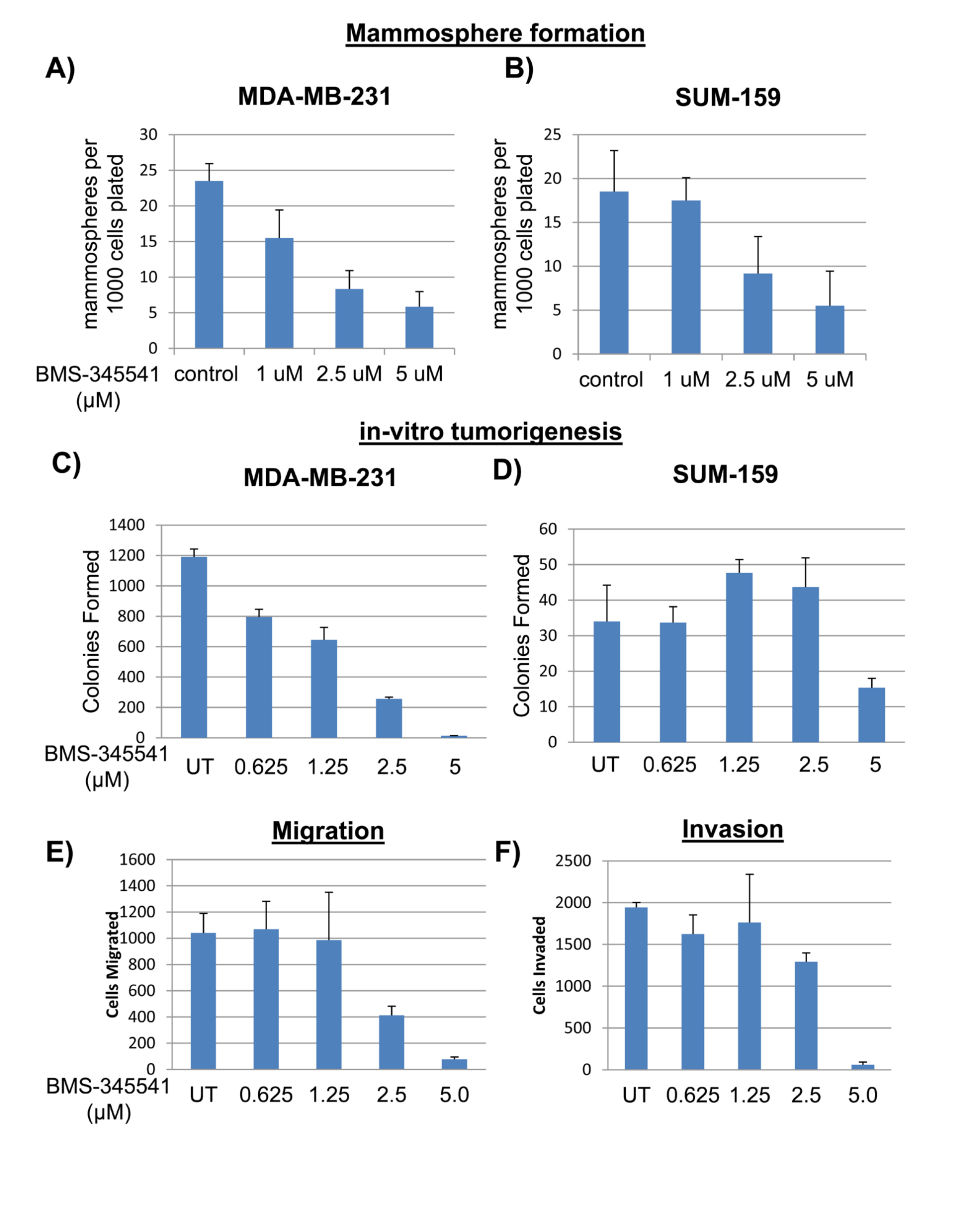
BMS-345541 inhibits function of BCSCs **A**. The bar graphs represent the number of mammospheres formed per 5000 MDA-MB-231 (A) or SUM159 **B**. cells seeded in low-adherent conditions with varying concentrations of BMS-345541 for 2 wk. **C**. and **D**. MDA-MB-231 (C) or SUM159 (D) cells were plated into soft agar with varying concentrations of BMS-345541. After 3 weeks of incubation, colonies were fixed with MTT and then counted using an automated colony counter. **E**. SUM159 cells were plated into trans-well migration plates with varying concentrations of BMS-345541. After 6 h, the membrane was fixed and stained, and then cells were counted. **F**. SUM159 cells were plated into trans-well invasion plates with matrigel; the media contained varying concentrations of BMS-345541. After 24 h, the membrane and Matrigel were removed, and the cells were counted.

To investigate whether BMS-345541 could inhibit the behavior of BCSCs, we treated MDA-MB-231 and SUM159 cells with various concentrations of BMS-345541 and tested their ability to endure anchorage-independent growth in soft agar and as mammospheres, and also their ability to undergo migration and invasion *in vitro*. BMS-345541 treatment inhibited anchorage-independent mammosphere formation in both MDA-MB-231 (Figure 3A) and SUM159 cells (Figure 3B) by 4- to 5-fold compared with the vehicle control. Soft agar colony assay revealed that BMS-345541 inhibited growth by > 98% in MDA-MB-231 cells and by > 60% in SUM159 cells, suggesting that NFκB-mediated signaling is required for BCSC tumorigenic qualities (Figure 2C and 2D). Interestingly, the migration assay revealed that BMS-345541 treatment inhibited this activity by SUM159 by approximately 10-fold (Figure 2E). The matrigel assay using trans-well chambers revealed that BMS-345541 treatment triggered a concentration-dependent inhibition in invasion of SUM159 cells by > 95% in (Figure 2F). These results suggest that BMS-345541 could inhibit NFκB, which was seemingly coupled to the tumorigenic characteristics of BCSCs.

### BMS-345541 inhibits breast tumor growth and metastases *in vivo*

**Figure 4:**
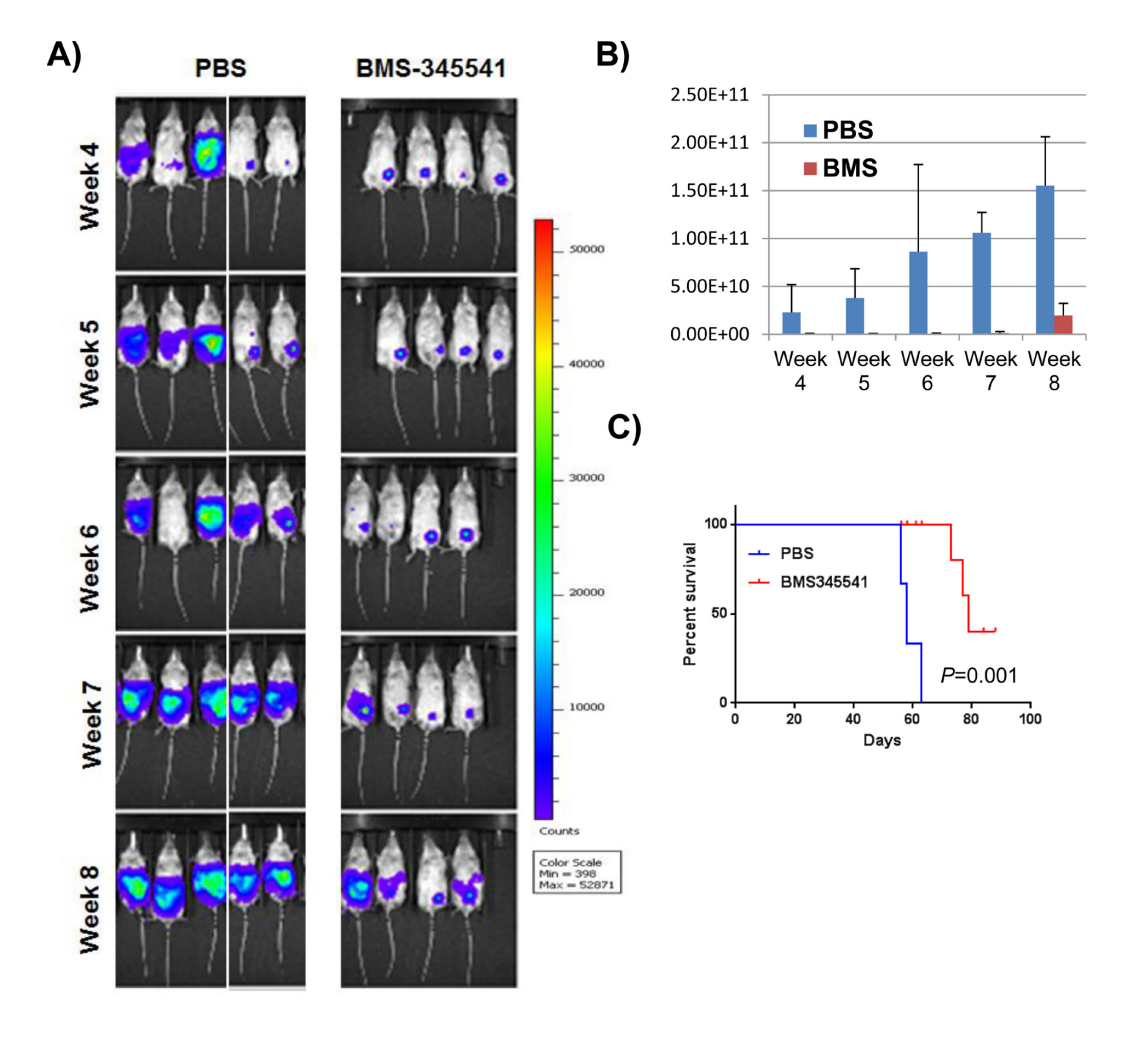
BMS-345541 reduces the rate of tumor growth and increases survival in tumor-bearing mice **A**. GFP/luciferase-expressing MDA-MB-231 cells were implanted into the mammary fat pads of mice (*n* = 14), who were then split into two treatment groups (Seven mice per group); one group was treated with BMS-345541 (25 mg/kg for three treatments per wk for 4 wk), while the other group was treated with control. The images show the luciferase activity in the mice over time. **B**. The bar graph represents the luminescence levels in the two groups of tumor-bearing mice. The *y*-axis represents the number of photons emitted. **C**. Survival analysis: Ten mice received MDA-MB-231 cells (1 × 10^6^ cells per mouse) in the mammary fat pads. One group was treated with BMS-345541, while the other group was treated with PBS as a control (three injections per week for 1 week). The survival curves were generated using Prism software (GraphPad Software, San Diego, CA).

To investigate the effect of BMS-345541 on *in vivo* tumor growth, 1 × 10^6^ MDA-MB-231 cells expressing GFP and firefly luciferase were implanted in mammary fat pads of NSG mice (*n* = 14). Upon palpable tumor formation (in roughly 2 wk), the mice were divided into two groups (n = 7 per group) and treated with PBS or 25 mg/kg BMS-345541 for 3 d per week for 4 wk via intra-peritoneal (IP) injections. Tumor growth was measured on a weekly basis by bioluminescence imaging (Figure 4A). We found that BMS-345541 treatment reduced tumor growth 2- to 3-fold compared to the control (Figure 4B). In addition, BMS-345541 treatment increased median survival of the mice by > 2 wk compared with the control (Figure 4C, 78 d for the BMS-345541-treated group vs. 58 d for the control-treated group; *P* < 0.002), suggesting that the inhibition of NFκB by BMS-345541 could inhibit breast tumor growth *in vivo* presumably by blocking BCSC function.

**Figure 5:**
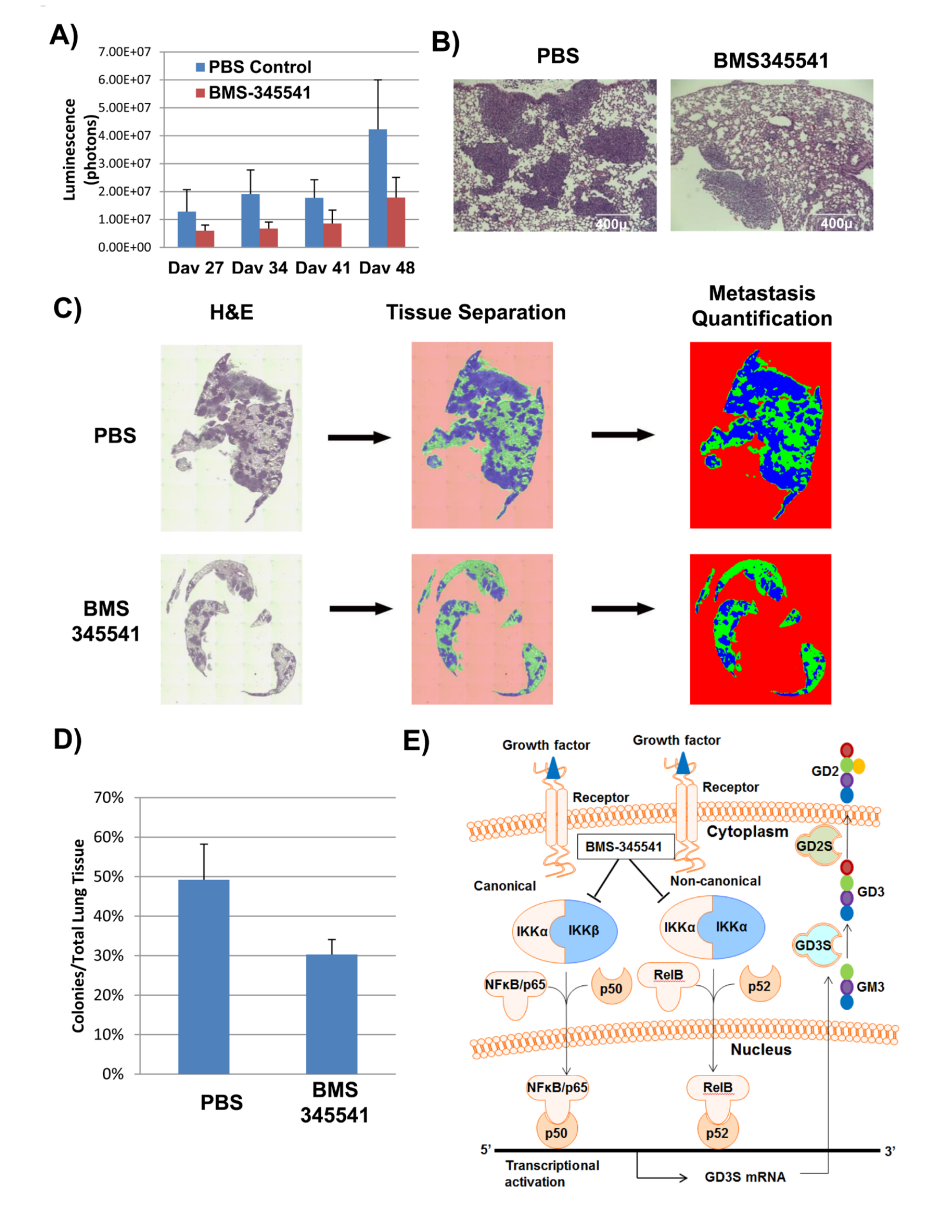
BMS-345541 inhibits cancer metastasis in *in vivo* settings **A**. GFP/luciferase-expressing MDA-MB-231 cells were injected into the tail veins of NSG mice (*n* = 10) in an experimental metastatic model. The mice were then split into two treatment groups; one group (5 mice per group) was treated with BMS-345541, while the other group was treated with PBS. The bar graph represents the luciferase activity of the groups. **B**. H and E staining of lung tissues derived from mice in experiment described in Figure 5A. The sections are derived from mice on d 34 after tumor implantation. **C**. and **D**. To quantitate the amount of metastasis in each group, lungs derived from treated and untreated groups on d 34 were stained with hematoxylin and eosin, and the sections were scanned using EVOS-FL auto microscope and the metastasis was quantitated using inForm software (PerkinElmer). **E**. The image illustrates the mechanism of action for BMS-345541, an IKK inhibitor. Through inhibition of IKK activity, IκB fails to get phosphorylated, leading to the inhibition of NFκB translocation across the nuclear membrane and inhibition of GD3S and GD2 expression.

To investigate whether BMS-345541 inhibits breast cancer metastases, we used an experimental metastasis model and intravenously implanted MDA-MB-231 cells expressing GFP and firefly luciferase in NSG mice (*n* = 10). One d after implantation, bioluminescence imaging was performed to ensure that all the mice had similar engraftment. Three d after implantation, the mice were divided into two groups and started on treatment with PBS or BMS-345541 (25 mg/kg) for 3 d per wk for 4 wk via intravenous injections. Tumor metastases were measured weekly by bioluminescence imaging. We found that BMS-345541 treated mice showed a reduction of reduced total bio-luminescence flux of 2- to 3-fold compared to the controls (Figure 5A). Immunohistochemical analysis by hematoxylin-eosin staining of lung tissues revealed that the BMS-345541-treated group had 3- to 4-fold fewer metastases than the PBS-treated group (Figure 5B). In addition, the size of the metastases was also significantly smaller for the mice treated with BMS-345541 (Figure 5C), indicating that BMS-345541 inhibits GD2^+^ BCSC function and thereby inhibits breast cancer metastases.

## DISCUSSION

We found that inhibition of NFκB signaling using the IKK blocker BMS-345541 suppressed GD2^+^ cell number apparently by inhibiting GD3S expression. In addition, BMS-345541 inhibited the tumorigenic function of BCSCs *in vitro*, and also inhibited *in vivo* tumor growth and metastases in immunodeficient mice implanted with BCSCs, suggesting a critical role of NFκB signaling in BCSC function.

We have previously reported that the ganglioside GD2 identifies BCSCs and that GD3S regulates GD2 expression in these cells [5]. In addition, treatment with the anti-inflammatory and anti-cancer drug triptolide dramatically inhibited GD2^+^ cells by inhibiting GD3S in MDA-MB-231 and SUM159 cells [5, 6], but the mechanism of action was not known. Triptolide has been reported to inhibit NFκB signaling in T-lymphocytes [17]. Previous studies also report the regulation of breast cancer stem cells by NFκB signaling, but did not measure NFκB activation exclusively in BCSCs [14, 18]. In this report, we show that NFκB signaling is activated in GD2^+^, but not in GD2^-^, breast cancer cells. In addition, we identify BMS345541 as potential tool for modulating GD3S and GD2 by interrupting NFκB signaling.

Activation of NFκB signaling in breast cancer has been reported by several investigators [11–13]. Singh et al. first showed NFκB activation in estrogen receptor-negative and Her2^+^ breast tumors and suggested NFκB as a therapeutic target [12]. Cogswell *et al*. reported selective activation of the NFκB subunit NFκB2/p52 in human breast cancer [13]. In addition, our group previously reported that over-expression of the IκB super-repressor inhibited GD3S expression in MDA-MB-231 and human mammary epithelial cells expressing Ras oncogene (HMLER). Kang *et al*. first identified the GD3S promoter sequence (-1146 to -646) in Jurkat T-cells and reported several transcription factors, including cETS1, CREB, AP-1, and NFκB binding sites, in the GD3S promoter sequence [19]. Deletion of NFκB binding sites inhibited Fas-induced GD3S expression, suggesting that GD3S and GD2 expression may depend on NFκB signaling. Reports from Bobowski *et al*. that estradiol inhibits GD3S expression in breast cancer cells by preventing NFκB to the GD3S promoter [20] further strengthens our current finding that NFκB regulates GD3S and GD2 expression.

Recent reports also suggest that the NFκB pathway is highly activated in cancer-initiating cells, specifically in BCSCs [18]. Yamamoto *et al*. reported that NFκB non-cell-autonomously regulates cancer stem cell populations in the basal-like breast cancer subtype [14]. The authors found that NFκB regulates JAG-1 expression and that the NIK-NFκB-JAG1-NOTCH axis regulates the BCSC population. Moreover, Park *et al*. reported that NFκB signaling promotes osteolytic bone metastasis by inducing osteoclastogenesis via granulocyte macrophage colony-stimulating factor [21]. In this report, the authors found that granulocyte macrophage colony-stimulating factor is a key target of NFκB and mediates osteolytic bone metastasis of breast cancer by stimulating osteoclast development. Similarly, our metastases data indicate that BMS-345541 treatment inhibits breast cancer metastases in mice by inhibiting GD2^+^ BCSCs, suggesting a critical role of NFκB signaling in breast cancer metastasis.

Kendellen *et al*. reported that both canonical and non-canonical NFκB pathways promote BCSCs. Knockdown of IKKα or IKKβ inhibited epithelial-mesenchymal transition marker expression [22]. We tried several inhibitors, including JSH-23, wedelolactone, and BMS-345541, to inhibit NFκB activity and its downstream targets; only BMS-345541 successfully inhibited GD3S and GD2 expression in a concentration-dependent manner (Figure 2). BMS-345541 targets both IKKα (half maximal inhibitory concentration: 4.2 μM) and IKKβ (half maximal inhibitory concentration: 0.4 μM) [23], which could be why BMS-345541 was more effective in inhibiting GD3S expression than the other NFκB inhibitors. A low effective dosage, high specificity for GD2^+^ BCSCs, inhibition of BCSC growth *in vivo* and *in vitro* make BMS-345541 a potential therapeutic tool for basal-like or triple-negative breast cancer subtypes and suitable for clinical trials.

In conclusion, our data indicate that NFκB-mediated signaling is activated in GD2^+^ BCSCs and regulates GD3S expression. BMS-345541 appears to inhibit canonical and non-canonical NFκB signaling by blocking IKKα and β in GD2^+^ cells inhibiting their ability to form tumors. In combination with conventional chemotherapeutic agents, BMS-345541 could be a potential therapeutic tool in the treatment of metastatic breast cancer.

## MATERIALS AND METHODS

### Cell culture

Human breast cancer cell lines MDA-MB-231 and SUM159 were cultured according to American Type Culture Collection recommendations.

### Flow cytometry analysis and fluorescence-activated cell sorting (FACS)

Single-color staining of MDA-MB-231 and SUM159 cells was performed as described previously [24]. Briefly, ~1 × 10^6^ cells were washed twice with phosphate-buffered saline (PBS) containing 1% fetal bovine serum (FACS buffer), incubated with anti-GD2 antibody conjugated with allophycocyanin (APC, BioLegend, San Diego, CA) for 30 minutes on ice, and washed in FACS buffer containing 4’,6-diamidino-2-phenylindole (DAPI) (0.5 mg/ml). After being washed, the cells were analyzed with an LSR II flow cytometer (BD; Franklin Lakes, NJ), and the cytometry data were analyzed using FlowJo software. At least 1 × 10^4^ events were measured per sample. FACS was performed to isolate GD2^+^ and GD2^-^ cells from MDA-MB-231 and SUM159 cells. Cells were stained with anti-GD2 antibody as described above, and sorting was performed on a FACSAria II cell sorter (BD Biosciences, Franklin Lakes, NJ). The absolute number of cells was found using TruCount counting beads (BD Biosciences) per the manufacturer's instructions.

### Proteomic analysis

To investigate signaling pathways activated in BCSCs, GD2^+^ and GD2^-^ MDA-MB-231 and SUM159 cells underwent FACS and were analyzed on an antibody microarray (Kinexus, Vancouver, Canada). Briefly, the cell lysates from GD2^+^ and GD2^-^ cells were labeled with fluorescent probes and applied to glass slides coated with 850 validated antibodies against various total proteins and phosphoproteins per the manufacturer's instructions. After washes, the staining was developed and analyzed on a phosphoimager. The Z-ratio was calculated as described previously [15], and proteins with differential expression or phosphorylation between GD2^+^ and GD2^-^ cells were identified. To understand the functional significance of and signaling pathways associated with GD2 and GD3S expression, we analyzed differentially expressed or activated proteins using Ingenuity Pathway Analysis software (Ingenuity Systems, Redwood City, CA).

### Generation of stable IKKα knockdown breast cancer cells

We used pLKO lentiviral vectors expressing shRNA against IKKα (Catalog # RHS3979-201732780; GE Dharmacon, Lafayette, CO) to knockdown IKKα in MDA-MB-231 cells. Lentiviral particle expressing IKKα shRNA were generated using packaging vectors as described previously [5]. The viral supernatant was then incubated with MDA-MB-231 cells for 48 h and the transduced clones were selected by incubating the cells in puromycin (ThermoFisher Scientific), containing cell culture medium at 2 µg/ml concentration.

### Western blotting

Western blotting was performed as described before [25]. Briefly, 1 million cells were plated into 100-mm-wide tissue culture dishes and incubated at 37°C and 5% CO_2_ for 24 hours to allow for adjustment and adherence. The cells were treated with PBS or BMS-345541 at various concentrations, including 0.0625, 1.25, 2.5, and 5 µM, for 24 hours. Cells were subjected to lysis at a density of 3 × 10^5^/50 µl in protein lysis buffer (G-Biosciences, St. Louis, MO), mixed with 2× laemmli buffer (Bio-Rad, Hercules, CA) at 1:1 ratio, and supplemented with a protease inhibitor cocktail (Roche Diagnostics, Risch-Rotkreuz, Switzerland). Cell lysates were loaded onto 10% polyacrylamide gel (Bio-Rad) for electrophoresis, and then the proteins were transferred to Immobilon-FL membranes (EMD Millipore, Billerica, MA). The membranes were incubated with rabbit anti-pNFκB or rabbit anti-RelB (both from Cell Signaling Technology, Danvers, MA) at a 1:500 or 1:1000 dilution and with mouse anti-glyceraldehyde 3-phosphate dehydrogenase (GAPDH) antibodies at a 1:10,000 dilution overnight at 4°C. The membranes were then washed and incubated with donkey anti-mouse immunoglobulin G antibody conjugated with Alexa Fluor 700 and donkey anti-goat antibody conjugated with Alexa Fluor 800 (both from Thermo Fisher Scientific, Waltham, MA). The membranes were washed again and scanned using the Odyssey fluorescence imaging system (LI-COR Biosciences, Lincoln, NE). For analysis of IKKα knockdown cells, the membranes were incubated with anti-IKKα antibody (Catalog# SC-7219, Santa Cruz Biotechnology) or GD3S (Catalog# HPA026775, Sigma-Aldrich) at 1:100 and 1:200 concentrations respectively. GAPDH served as a loading control.

### Real-time reverse transcription polymerase chain reaction (RT-PCR)

A total of 5 × 10^5^ MDA-MB-231 or SUM159 breast cancer cells were incubated with BMS-345541 at concentrations of 0.625, 1.25, 2.5, and 5 μM or the control for 24 h at 37°C in six-well cell culture dishes. Real-time RT-PCR was performed using TaqMan gene expression assays from Applied Biosystems (Carlsbad, CA) as described previously [26]. The assay for GD3S was Hs00268157, and the assay for GAPDH was Hs02758991.

### Mass cytometry

To compare activation of cell signaling proteins between GD2^+^ and GD2^-^ MDA-MB-231 cells, Time-of-flight mass cytometry (CyTOF), a novel technology combining atomic mass spectrometry and flow cytometry, was performed as described before [27]. Briefly, the cells were fixed in 1.6% formaldehyde to stop signal transduction and place the cells in stasis. We then incubated the cells with extracellular anti-GD2 antibody (clone 14.G2a; BD Biosciences) conjugated with samarium (isotopic mass: 152). Once extracellular antibody binding was complete, the cells were fixed and permeabilized with 50% methanol and then were incubated with an intracellular antibody cocktail, which consisted of anti-pNFκB (p65)-Sm149, anti-pPI3K(Y607)-Gd158, and anti-pmTOR(S248)-Dy164. Anti-pPI3K(Y607)-Gd158 and anti-pmTOR(S248)-Dy164 were kindly gifted by Dr. Scott Tanner from the University of Toronto, Canada. After extracellular and intracellular binding, we stained the cells with iridium dye (DNA intercalator-Rh103; Fluidigm) overnight to prepare for CyTOF. PBS with 0.5% bovine serum albumin was used as a wash and incubation buffer throughout, as it acts as a carrier protein, improving antibody binding and aiding with washing. The cells were analyzed on CyTOF mass cytometry (Fluidigm, South San Francisco, CA). The data were saved in Flow Cytometry Standard 3.0 format and analyzed by spanning-tree progression analysis of density-normalized events (SPADE) software, a novel tool for analyzing complex data sets involving multiple parameters [28].

### Mammosphere assay

To investigate the effect of BMS-345541 on the mammosphere formation potential of MDA-MB-231 and SUM159 cells, 1 × 10^2^ or 1 × 10^3^ cells were plated in ultra-low attachment 24-well dishes containing mammosphere growth medium (Stemcell Technologies, Vancouver, BC, Canada). The cells were treated with or without BMS-345541 at concentrations of 0.625, 1.25, 2.5, and 5 μM. After 10 to 12 d of culture at 37°C in humidified air containing 5% CO_2_, the resulting mammospheres were counted using a microscope.

### *In vitro* tumorigenesis assay

Two and half milliliters of liquefied solution of 1% low-melting agarose (Thermo Fisher Scientific, Fair Lawn, NJ) in DMEM medium with 10% FCS with appropriate drug concentrations (BMS-345541 at 0, 0.625, 1.25, 2.5, and 5 μM) was added to each well in a six-well plate. After the plates were cooled at 4°C for 1 hour, an additional 2.5 ml of 0.5% agarose, complete media solution, the same drug concentrations as above, and 5000 MDA-MB-231 or SUM159 cells were added to each well. The plates were then cooled at 4°C for 30 min and then incubated at 37°C and 5% CO_2_ for 3 wk (100 µl of media were added weekly to avoid drying of the soft agar gels). The resulting colonies were fixed and stained using 200 µl/well of 1 mg/ml of 3-(4,5-dimethylthiazol-2-yl)-2,5-diphenyltetrazolium bromide (MTT) (Alfa Aesar, Heysham, United Kingdom). The colonies were then counted using an automated colony counter (Oxford Optronix, Abingdon, United Kingdom).

### Migration assays

Migration assays were performed using 24-well trans-well chambers as described previously [5]. Membrane inserts with 8-µm pores (Corning, Kennebunk, ME) were used to test the effect of BMS-345541 on cellular migration. The bottom chamber was filled with complete cell culture medium with 10% FCS. Added to the upper chamber were 3 × 10^5^ MDA-MB-231 or SUM159 cells that were suspended in 200 μl of Dulbecco's Modified Eagle Medium containing 1% FCS and BMS-345541 at concentrations of 0.625, 1.25, 2.5, and 5 μM and the control. The chambers were incubated at 37°C and 5% CO_2_ for 6 h. The cells that migrated to the other side of the membrane were stained with DAPI (Life Technologies, Carlsbad, CA) and counted under the EVOS-FL automated microscope (Thermo Fisher Scientific).

### *In vitro* matrigel invasion assay

*In vitro* migration and invasion assays were performed using 24-well Biocoat Matrigel Invasion Chambers (Corning) containing BD Falcon Cell Culture Inserts with a polyethylene terephthalate membrane (8-µm-diameter pore size) treated with Matrigel Matrix (BD Biosciences) as described previously [5]. Briefly, cell suspensions of MDA-MB-231 and SUM159 cells were placed in the Matrigel-coated upper chamber. After incubation at 37°C for 24 h in a 5% CO_2_ incubator, the residual cells on the upper surface of the filter were completely removed with cotton swabs. The membranes were then stained, and the cells were counted.

### *In vivo* tumor growth

All experiments involving animals were approved by and conducted in accordance with the Institutional Animal Care and Use Committee of The University of Texas MD Anderson Cancer Center. GD2^+^ and GD2^-^ MDA-MB-231 cells underwent FACS using a FACSAria II cell sorter (BD). The GD2^+^ and GD2^-^ MDA-MB-231 cells were then transplanted subcutaneously into non-obese diabetic/severe combined immunodeficiency (NSG) mice at concentrations of 10,000, 1000, or 100 cells per site to observe tumor growth. Two injection sites were used per mouse, and five mice were used in each group. The tumors were identified by palpation after 2 wk immediately before the mice were divided into groups. The tumors were treated with BMS-345541 or a control and monitored by bioluminescence imaging. In a similar experiment, MDA-MB-231 cells were subcutaneously transplanted (1 × 10^6^ per site) into NOD/SCID mice to observe the cells’ tumor initiation potential. In both experiments mice were killed after tumors reached a diameter of 1.5 cm, in accordance with the institutional guidelines.

### *In Vivo* metastasis assay

All experiments involving animals were approved by and conducted in accordance with the Institutional Animal Care and Use Committee of The University of Texas MD Anderson Cancer Center. 500 thousand MDA-MB-231 GFP/Luciferase cells were injected into the tail veins of 20 NSG mice. The mice were then imaged on d 1 and d 6 using the IVIS live bioluminescence imager. After ensuring equal engraftment, the mice were treated twice a week starting on d 7. On d 35, lungs were retrieved, fixed, and stained using hematoxylin and eosin. The slides then imaged using the EVOS (ThermoFisher) imaging system. Whitefield images were sectioned into healthy and metastatic lung colonies using inForm (PerkinElmer) analysis. The sectioned images were converted into simple Red, Green, Blue images. Using Photoshop (Adobe, San Jose, CA), pixels were counted and used to calculate the percentage of metastatic colonies per lung tissue.

### Statistical analyses

Unless otherwise indicated, data are mean ± standard error of the mean. The statistical significance of tumor growth was determined by a two-way analysis of variance for repeated measures. All other group differences were evaluated by two-tailed unpaired Student t*-*tests. Survival data were analyzed using Kaplan-Meier log-rank tests. A *P* < 0.05 was considered statistically significant.

## SUPPLEMENTARY MATERIALS FIGURES AND TABLES


