# Supplementary material for: IKK inhibition by BMS-345541 suppresses breast tumorigenesis and metastases by targeting GD2+ cancer stem cells
Source: Oncotarget. 2017 Mar 16;8(23):36936–49. doi: 10.18632/oncotarget.16294 (PMC5514883; doi:10.18632/oncotarget.16294)
Supplement: Supplementary file 1 [file oncotarget-08-36936-s001.pdf]

IKK inhibition by BMS-345541 suppresses breast tumorigenesis and metastases by targeting GD2^{+} cancer stem cells

Supplementary Material

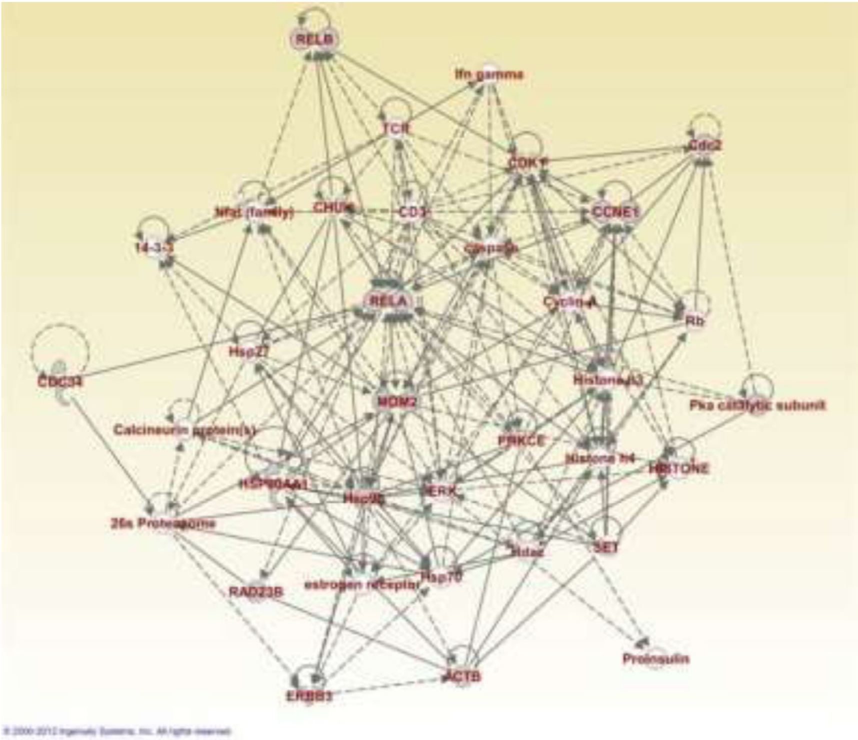

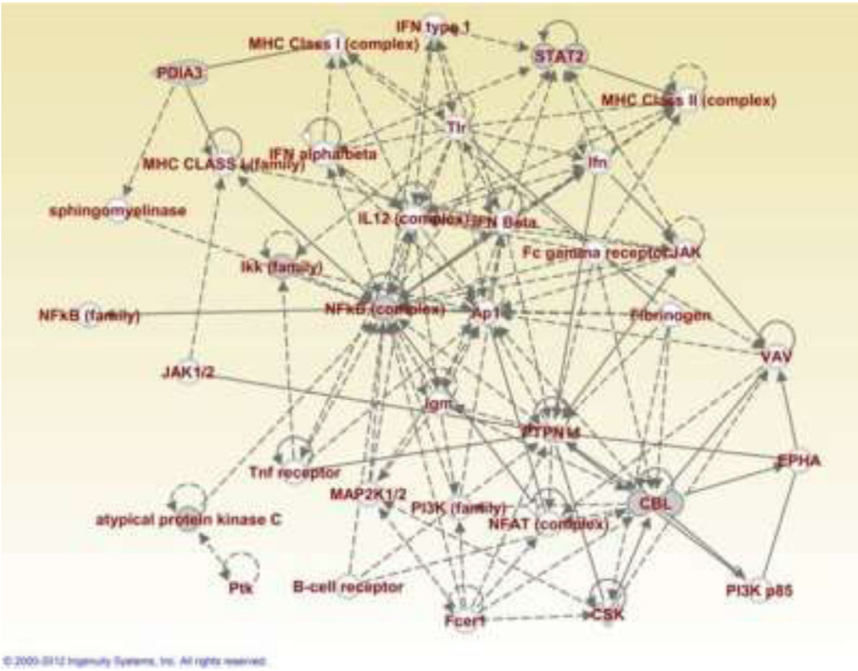

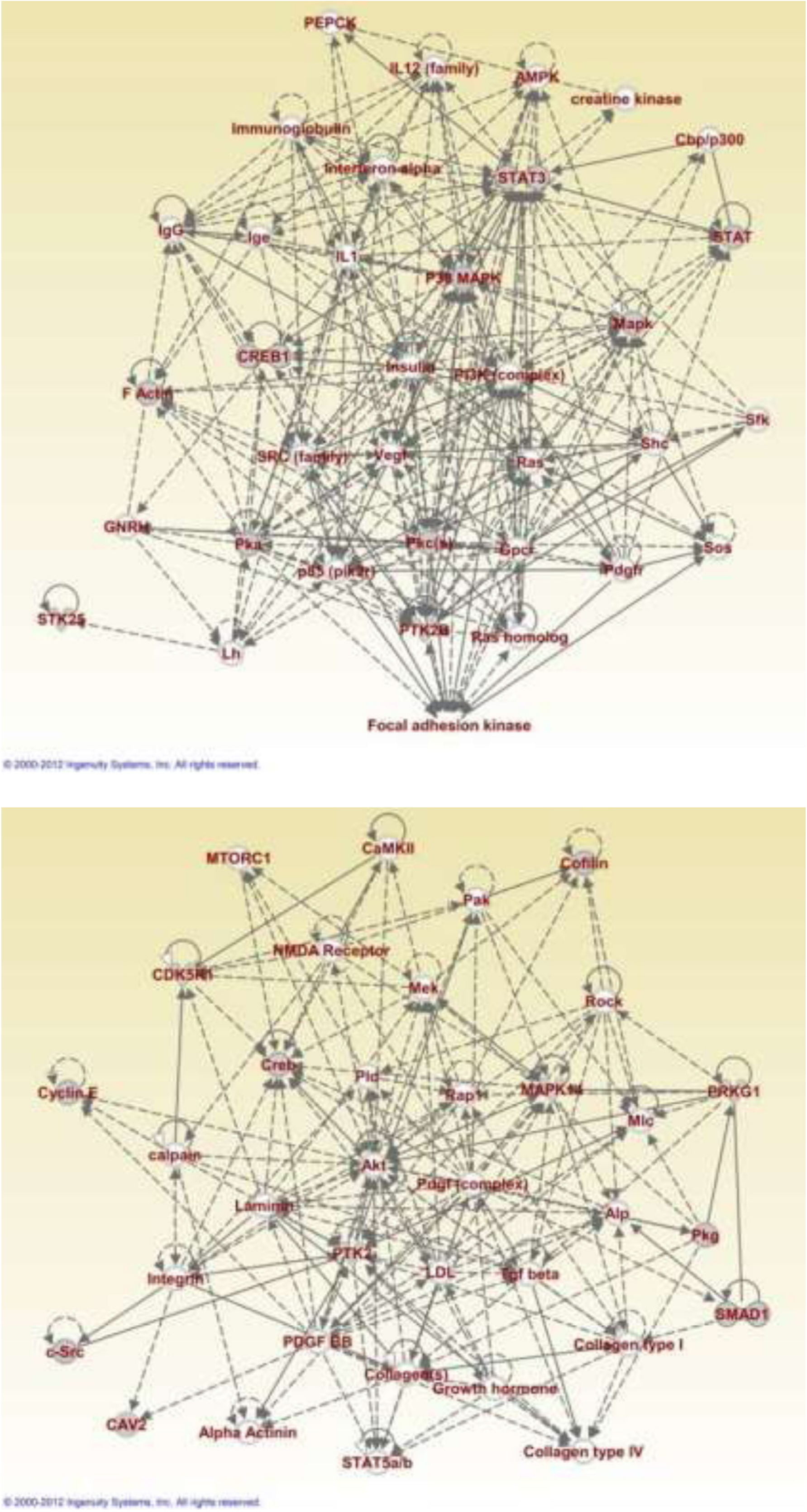

© 2000-2012 Ingenuity Systems, Inc. All rights reserved.

Supplementary Figure 1: Ingenuity pathway analysis tool revealed the activation of NFκB, STAT3, focal adhesion kinase (FAK), p53, platelet-derived growth factor β (PDGFβ), and p38 MAPK signaling pathways, among others, in the GD2^{+} cells.

Supplementary Table 1: *Proteins up- or down-regulated in GD2^{+} compared with GD2^{-} cells.* Protein lysates from MDA-MB-231 cells were analyzed on antibody microarrays (Kinexus). Proteins are grouped and highlighted as being differentially activated (pink) or suppressed (blue), respectively.

Target Protein Name	Phospho Site (Human)	Full Target Protein Name	Z-ratio (MDA231 -GD2+, MDA231-GD2-)
VHR	Pan-specific	Dual specificity protein phosphatase 3	1.99
FAK	S910	Focal adhesion protein-tyrosine kinase	1.98
CDK6	Y13	Cyclin-dependent protein-serine kinase 6	1.93
ERP57	Pan-specific	ER protein 57 kDa (protein disulfide isomerase-associated 3; 58 kDa glucose regulated protein)	1.80
anti-actin	Pan-specific	Actin	1.78
hHR23B	Pan-specific	UV excison repair protein RAD23 homolog B	1.60
PKCq	Pan-specific	Protein-serine kinase C theta	1.57
Csk	Pan-specific	C-terminus of Src tyrosine kinase	1.55
4E-BP1	T45	Eukaryotic translation initiation factor 4E binding protein 1 (PHAS1)	1.47
PKA Ca/b	Pan-specific	cAMP-dependent protein-serine kinase catalytic subunit alpha/beta	1.46
4E-BP1	S65	Eukaryotic translation initiation factor 4E binding protein 1 (PHAS1)	1.44
NFKB p65	S529	NF-kappa-B p65 nuclear transcription factor	1.34
Acetylated Lysine	Pan-specific	Acetylated Lysine	1.29
STAT3	Pan-specific	Signal transducer and activator of transcription 3 (acute phase response factor)	1.28
CREB1	S129+S133	cAMP response element binding protein 1	1.24
Acetylated Lysine	Pan-specific	Acetylated Lysine	1.19
PTP1D	Pan-specific	Protein-tyrosine phosphatase 1D (SHP2, SHPTP2, Syp, PTP2C)	1.13
PKCl/i	Pan-specific	Protein-serine kinase C lambda/iota	1.08
ErbB3	Y1328	Tyrosine kinase-type cell surface receptor HER3	1.08
Cofilin 1	S3	Cofilin 1	1.06
FAK	S722	Focal adhesion protein-tyrosine kinase	1.06
I2PP2A	Pan-specific	Protein SET	1.04
Cyclin G1	Pan-specific	Cyclin G1	-1.00
PP2Cd	Pan-specific	Protein-serine phosphatase 2C - catalytic subunit - delta isoform	-1.01
PKCm (PKD)	S910	Protein-serine kinase C mu (Protein kinase D)	-1.03

CDK2	Pan-specific	Cyclin-dependent protein-serine kinase 2	-1.03
DNAPK	Pan-specific	DNA-activated protein-serine kinase	-1.06
IkBa	Pan-specific	Inhibitor of NF-kappa-B alpha (MAD3)	-1.09
CDK8	Pan-specific	Cyclin-dependent protein-serine kinase 8	-1.10
Histone H3	T4	Histone H3.3	-1.12
CDK1 (CDC2)	Pan-specific	Cyclin-dependent protein-serine kinase 1	-1.12
PKCg	Pan-specific	Protein-serine kinase C gamma	-1.14
JNK1/2/3	Pan-specific	Jun N-terminus protein-serine kinase (stress-activated protein kinase (SAPK)) 1/2/3	-1.15
PAK2	Pan-specific	p21-activated kinase 2 (gamma) (serine/threonine-protein kinase PAK 2)	-1.16
FKBP52	Pan-specific	FK506-binding protein 4	-1.20
CDK6	Pan-specific	Cyclin-dependent protein-serine kinase 6	-1.21
CDK1 (CDC2)	Pan-specific	Cyclin-dependent protein-serine kinase 1	-1.29
IkBa	Pan-specific	Inhibitor of NF-kappa-B alpha (MAD3)	-1.35
Histone H1	phospho CDK1 sites	Histone H1 phosphorylated	-1.35
Cdc25C	Pan-specific	Cell division cycle 25C phosphatase	-1.37
Hsc70	Pan-specific	Heat shock 70 kDa protein 8	-1.40
PKCh	T655	Protein-serine kinase C eta	-1.48
CDK1/2	T14+Y15	Cyclin-dependent protein-serine kinase 1/2	-1.52
Histone H2B	S15	Histone H2B	-1.68
PKBa (Akt1)	Pan-specific	Protein-serine kinase B alpha	-1.99
COT	Pan-specific	Osaka thyroid oncogene protein-serine kinase (Tpl2)	-2.48
